# Eradication of Bovine Viral Diarrhoea (BVD) in Cattle in Switzerland: Lessons Taught by the Complex Biology of the Virus

**DOI:** 10.3389/fvets.2021.702730

**Published:** 2021-09-07

**Authors:** Matthias Schweizer, Hanspeter Stalder, Anja Haslebacher, Martin Grisiger, Heinzpeter Schwermer, Elena Di Labio

**Affiliations:** ^1^Institute of Virology and Immunology, Bern, Switzerland; ^2^Department of Infectious Diseases and Pathobiology, Vetsuisse Faculty, University of Bern, Bern, Switzerland; ^3^Veterinary Office Canton Solothurn, Solothurn, Switzerland; ^4^Veterinärdienst der Urkantone, Brunnen, Switzerland; ^5^Federal Food Safety and Veterinary Office (FSVO), Bern, Switzerland

**Keywords:** pestivirus, bovine viral diarrhoea virus, border disease virus, eradication, sheep, molecular epidemiology, persistent infection, transient infection

## Abstract

Bovine viral diarrhoea virus (BVDV) and related ruminant pestiviruses occur worldwide and cause considerable economic losses in livestock and severely impair animal welfare. Switzerland started a national mandatory control programme in 2008 aiming to eradicate BVD from the Swiss cattle population. The peculiar biology of pestiviruses with the birth of persistently infected (PI) animals upon *in utero* infection in addition to transient infection of naïve animals requires vertical and horizontal transmission to be taken into account. Initially, every animal was tested for PI within the first year, followed by testing for the presence of virus in all newborn calves for the next four years. Prevalence of calves being born PI thus diminished substantially from around 1.4% to <0.02%, which enabled broad testing for the virus to be abandoned and switching to economically more favourable serological surveillance with vaccination being prohibited. By the end of 2020, more than 99.5% of all cattle farms in Switzerland were free of BVDV but eliminating the last remaining PI animals turned out to be a tougher nut to crack. In this review, we describe the Swiss BVD eradication scheme and the hurdles that were encountered and still remain during the implementation of the programme. The main challenge is to rapidly identify the source of infection in case of a positive result during antibody surveillance, and to efficiently protect the cattle population from re-infection, particularly in light of the endemic presence of the related pestivirus border disease virus (BDV) in sheep. As a consequence of these measures, complete eradication will (hopefully) soon be achieved, and the final step will then be the continuous documentation of freedom of disease.

## Pestiviruses in Their Host Population

**Figure 1:**
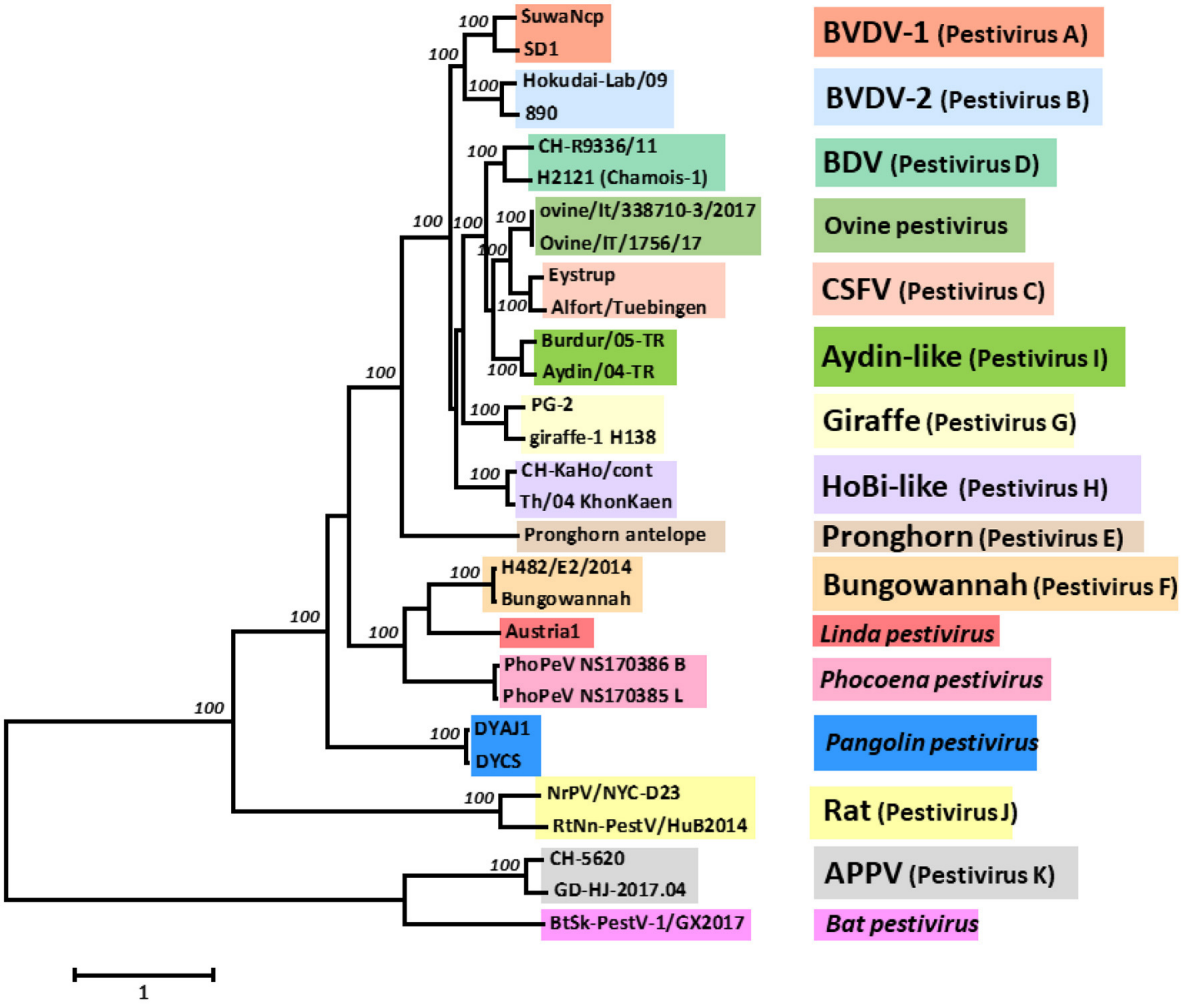
Phylogenetic analysis and classification of pestiviruses based on the nucleotide sequence of the entire open reading frame (ORF). The evolutionary history was inferred using the Maximum Likelihood method based on the General Time Reversible model (16), with the tree with the highest log likelihood being shown. A discrete gamma distribution was used to model evolutionary rate differences among sites. The percentage of replicate trees in which the associated taxa clustered together in the bootstrap test (100 replicates) are shown next to the branches (17), with only maximal values of 100 being shown. The tree is drawn to scale, with branch lengths measured in the number of substitutions per site. The analysis involved 29 nucleotide sequences. Codon positions included were 1st + 2nd + 3rd + non-coding. All positions with <95% site coverage were eliminated. There were a total of 9,912 positions in the final dataset. Evolutionary analyses were conducted in MEGA7 (18). The GenBank accession numbers of the sequences used are listed in Supplementary Table 1.

Pestiviruses have gained increased attention as several new species were discovered in recent years. Previously, the genus *Pestivirus* in the family *Flaviviridae* comprised the four species bovine viral diarrhoea virus (BVDV)-1 and−2, classical swine fever virus (CSFV) and border disease virus (BDV) from sheep (1, 2). In addition, several new members termed as “atypical pestiviruses” were not yet classified as species (3), e.g., giraffe pestivirus [(4, 5) and references therein], HoBi-like pestiviruses (6), Bungowannah virus (7), or a pestivirus from pronghorn antelopes (8). Recently, a number of new pestiviruses were described from a large variety of species, such as atypical porcine pestivirus (APPV) (9) and Linda virus (10) in pigs, phocoena pestivirus in harbour porpoise (11) and, outside the order Artiodactyla, pestiviruses in rats (12), bats (13), or pangolins (14) (Figure 1). Together with the fact that a number of pestiviruses exhibit a broad species tropism, it became evident that taxonomic classification of pestiviruses based on the host species they were isolated from was not feasible anymore. Therefore, a new nomenclature using alphabetic characters was proposed (15), such as *Pestivirus A, B, D*, and *H* for the widespread ruminant pestiviruses BVDV-1,−2, BDV, and HoBi-like, respectively, that this review will concentrate on.

Viruses use two different strategies to remain in their host population. On the one hand, the so called “hit & run” approach indicates that a primary host is infected for only a short duration requiring the virus to be rapidly transferred to the next host. Rabies virus, which ultimately kills the primary, transiently infected host, and influenza virus or the currently pandemic SARS-CoV-2 virus, all leave behind an at least partially immune host, and are typical examples of this approach. By contrast, the “infect & persist” (also called “hit & stay”) strategy indicates that the host is chronically or even lifelong infected, which mostly requires that the virus evolved sophisticated means to evade the host's immune system (19, 20). Well-known examples of the latter strategy are HCV, HIV, or herpesviruses.

The successful worldwide survival of BVDV (21) and other ruminant pestiviruses in their host population is based on the fact that they apply both strategies, i.e., transient and persistent infections (22). The latter is established upon foetal infection of pregnant cows within the first ~150 days of gestation with a non-cytopathic (ncp) biotype of BVDV. (i) This early time point of foetal infection prior to the development of adaptive immunity, (ii) the virus' ability to block the activation of the host's innate antiviral response, and (iii) the distinct epitheliochorial placentation of ruminants that does not allow the transfer of maternal antibodies, leads to virus-specific B- and T-cell immunotolerance and the birth of a persistently infected (PI) calf (23, 24). They might appear healthy, but respiratory symptoms are more common in young animals whereas enteric symptoms are observed more often in older animals (25). In addition, the PI calves are at risk of developing fatal Mucosal Disease (MD), where both, a cytopathic (cp) and an ncp, biotype can be isolated. A large variety of mutations in the viral RNA genome of the ncp biotype, such as nucleotide substitutions or recombination with viral or host RNAs, lead to the emergence of an antigenically homologous cp biotype [for review, see e.g., (26–28)]. The cp biotype of BVDV can only spread in its host in the absence of an immune response and, therefore, it can only occur and disseminate in PI animals that are immunotolerant to strains that are antigenically identical to the persisting virus. Due to its systemic spread, cp BVDV ultimately kills its PI host, and thus represents an evolutionary dead-end for such pestivirus mutants (26). Although epidemiologically irrelevant, the dramatic clinical picture of MD in the last phase of BVDV infection has great implications for animal welfare. In contrast to other persistent viral infections such as herpesviruses, PI animals produce neither a cellular nor a humoral immune response against the persisting virus strain and remain, therefore, antibody negative. The PI animals continue to shed large amounts of virus for life and remain a constant thread to spread the virus to naïve animals and represent the most important reservoir maintaining the virus in its host population.

In addition to this persistence, acute infection of adult, naïve cattle with either biotype of BVDV results in transient viremia that is often asymptomatic or accompanied by only mild diarrhoea or respiratory symptoms, but in rare cases, severe thrombocytopenia and haemorrhages might be observed (29). During acute infection lasting ~2 weeks, virus might be found in various secretions and, thus, might be further transmitted to new, susceptible hosts. However, transient infections on their own are not sufficient to sustain virus circulation for long periods in its host, with a possible exception in large herds [(30–33), and unpublished observation], which are rarely found in Switzerland. Nevertheless, transient infections might well-contribute to local transmissions bypassing the temporary absence of susceptible, pregnant animals, finally leading to the infection of naïve, pregnant animals, and to the re-emergence of new PI animals that are required for the long-term survival of this virus in its host population (23). Thus, ruminant pestiviruses are successfully using both infection strategies, i.e., infect & persist as well as hit & run, which has direct consequences on the implementation of BVD control programs, e.g., interpretation of antigen- and antibody tests, or the time span taken into account at contact tracing, as discussed in this review.

Ruminant pestiviruses have probably circulated for hundreds of years in their hosts (34, 35) causing large economic losses (36–39). To reduce this financial burden, several countries, or regions introduced control programmes to reduce or even eradicate BVDV from the cattle population (40–50). In this review, we portray the eradication scheme implemented in Switzerland in 2008 describing pros and cons of the strategy chosen and exemplify various hurdles that appeared on the way to a BVDV-free Swiss cattle population. With >99.5% of herds being BVDV-free, Switzerland almost achieved this goal, and the experiences gained in the last decade might provide useful information for veterinary authorities implementing new control programmes in other areas.

## Swiss Eradication Scheme

**Figure 2:**
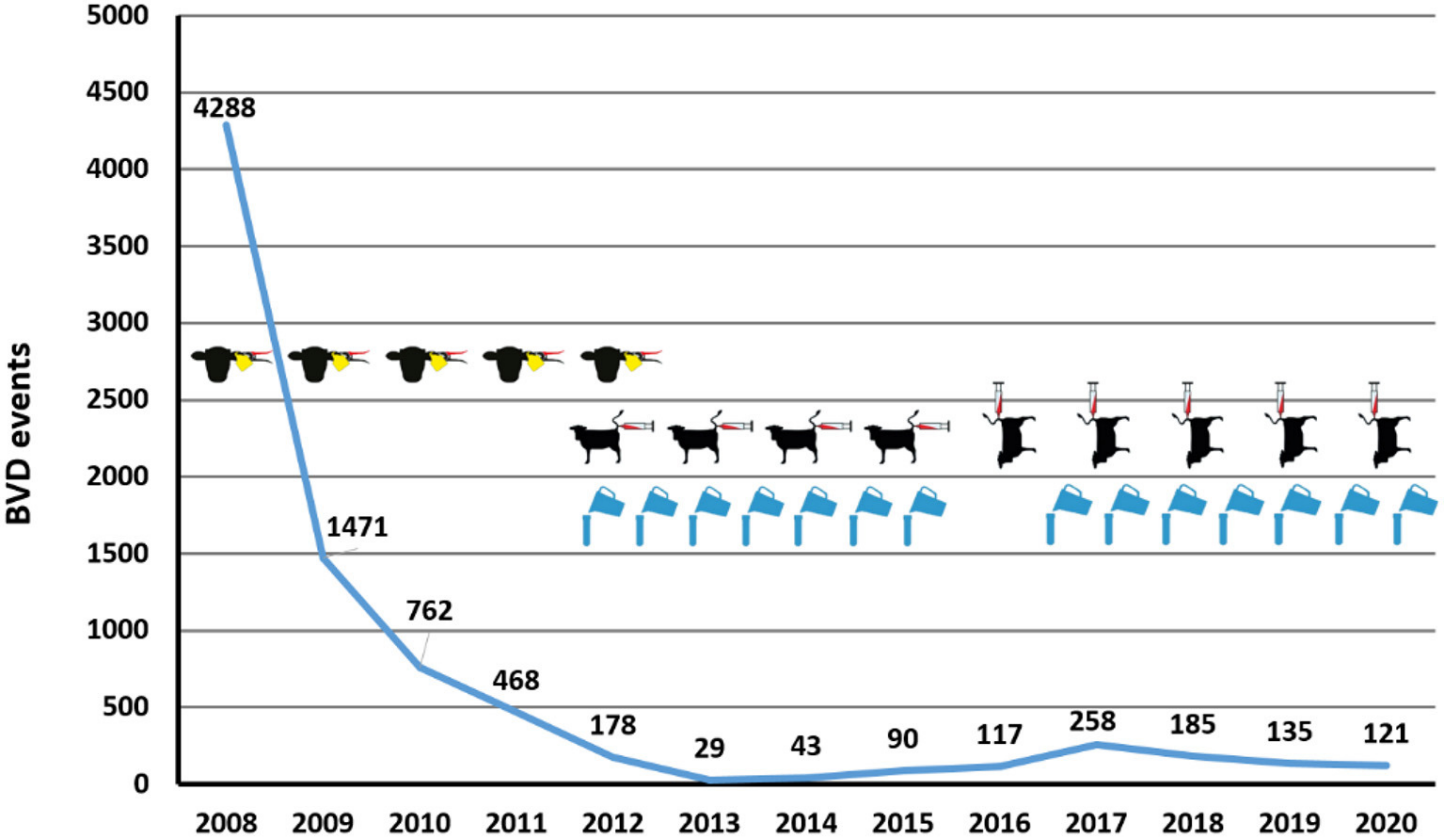
Number of yearly BVD events between 2008 and 2020 and method of BVD surveillance. The number of BVD events (detection of one or more PI animals in a previously BVD-free herd) (y-axis) per year (x-axis) are indicated (blue line with numbers indicated above). The surveillance mode applied in these years is indicated: (i) population screening in 2008 followed by yearly virological testing of all newborn calves (symbol: cattle head with ear tagging); (ii) spot tests by blood samples (symbol: cow with syringe) from living bovines sampled on farm (horizontal symbol) and mainly from slaughtered bovines (vertical symbol); (iii) bulk milk testing (symbol: blue flagon).

The entire cattle population in Switzerland comprises ~1.5–1.6 million animals, and annually, 600,000–700,00 calves are born (34). The disease costs due to BVDV were estimated between 9 and 16 million Swiss francs per year (51, 52), depending on the model applied and whether losses by transiently infected (TI) animals were included. This led the various breeding associations in Switzerland to demand eradication of BVDV from the Swiss cattle population. The Swiss BVD control programme started in 2008 and is based on the detection and elimination of every PI animal (Figure 2). The control programme was divided into three phases: (i) the initial phase when the entire cattle population was to be ear-notched and antigen tested, except pure fattening farms where animals only leave for slaughter, (ii) the calf phase with antigen testing of all newborn calves, and (iii) the surveillance phase with serological testing of disease-free herds via bulk milk in dairy herds and blood samples in beef herds (52–54). The latter phase meant that vaccination was prohibited from the outset. Two important additional basic principles were imposed that were deemed to be non-negotiable throughout the control scheme: First, cattle movements should not be hampered or only for a short time by testing or restrictions. This also required a simultaneous start to the national control programme in all cantons. Second, the case definition of an infected herd should be exclusively based on the detection of a PI animal. These directives would be expected to limit the economic burden posed by the eradication measures on the individual farms, and concomitantly, should increase the commitment of farmers to actively participate in the programme. Retrospectively, it might be questioned from an epidemiological viewpoint whether the decision not to regulate animal movements might have reduced the effectiveness of the control programme. Thus, animal movement was shown to have great importance for BVD control in Switzerland (55), and regulation of animal movement has been described as an important measure in the successful control programme in Sweden (56).

### 2008: Start With Virological Testing

In 2008, the whole cattle population was screened for the presence of PI animals, starting in spring with the animals that will spend the summer on common alpine pastures. In this initial screening, 0.8% of *all* bovines were virus-positive, and 20.0% of all herds had at least one virus positive animal (57). Subsequently, all newborn calves were tested for BVDV, either by antigen ELISA or by RT-PCR using ear notches, mostly taken by the farmer, or blood samples. Since autumn 2009, epidemiological investigations including contact tracing were required for every PI animal identified. The aim was to decrease the prevalence of PI animals close to zero within 3 years to be able to switch to surveillance based on serology (57, 58). Between 2008 to 2012, the proportion of all *newborn* calves being PI fell from 1.4% to <0.02% (59). In 2011, the situation was re-assessed, and it was concluded that the number of infected herds was still too high to start monitoring by serology. Concomitantly, the regional veterinary services were rather reluctant to abandon the simple and proven antigen testing scheme and to switch to the more complicated serological surveillance. In addition, owing to the high seroprevalence before the start of the eradication programme (60), the proportion of positive tank milk samples was assumed to be still too high to test the dairy herds accordingly. In addition, 55 PI animals that initially tested negative (“false negative”) were detected through epidemiological investigations until the end of 2010 (57), further implying that some gaps in the control scheme needed to be closed. Thus, the transition from virological to serological testing was postponed to 2012.

### 2012: Transition to Surveillance by Serology

In 2012, both testing schemes, i.e., testing all newborn animals for the presence of virus and herd testing for antibodies, were applied in parallel to gain more experience and to increase trust in the serological surveillance keeping a high commitment by all stakeholders to the control programme. Dairy herds were monitored by bulk milk serology, and the non-dairy herds by blood samples from a group of young cattle (so-called “young animal window” or “spot test”) (61, 62). The results of the bulk milk ELISA were categorised into 4 classes (see chapter “Detection of Antibodies”) according to their antibody level. All samples yielding an antibody result being categorised in class 3 and those from class 2 with an increase of the ELISA-PP value (percentage positivity value) of 4% or more compared to the previous test were regarded as “non-negative.” These definitions limited the number of herds with a positive (non-negative) result that were required to be examined by spot test. While the dairy herds were screened twice a year, the remaining herds were only monitored every third year, as sampling and analysing by spot test was the biggest cost drivers in serological surveillance. Data from 2012 indicated a lower risk of PI births in the non-dairy sector compared to dairy herds, justifying these different testing schemes retrospectively. Data obtained in this “transition year” indicated that the level of seropositivity appeared to be sufficiently low, and not least due to the high costs associated with virological testing of all calves (37, 52), the switch to exclusive serological surveillance was implemented in 2013 even if the PI prevalence of all newborn calves was still at ~0.02% (23, 59).

### 2013–2018: Antibody Surveillance

All dairy herds should be tested twice yearly and all non-dairy herds once every three year. The spot test should include at least five animals not <6 months of age and born after September 2009, or 10% of the stock in larger herds. Additional requirements for animals to be included in a spot test were (i) that they were born at least 1 month after the elimination of the last PI animal in the herd; (ii) that they stayed in the herd to be tested for at least 6 months, (iii) that they were not part of a herd containing a PI animal during and after its stay in that herd, and (iv) that the animals were not previously tested seropositive. Spot tests were also performed in dairy herds with a “non-negative” bulk milk result. The classification of a bulk milk test result as “non-negative” was adapted at the end of 2014, as the interpretation of the progression of the antibody titre in herds categorised as class 2 by the PP value between two samples was too complex. To simplify the interpretation, all samples in class ≥2 were defined as non-negative, despite this leading to an increase in positive results and a higher workload for the regional veterinary services. In the first 3 years of surveillance, samples for spot tests were taken on the farm by the cantonal veterinary services. This resulted in only about 80% of the non-dairy herds being sampled at least once in the 3 years. In addition, these herds were not tested uniformly in this period but most of them were tested in the last year. Thus, the increase in the case count 2013–2015 (Figure 2) could be at least partly attributed to the increased testing in non-dairy herds toward the end of this 3-year period.

In 2015, the sampling frequency for dairy herds was reduced to once per year, with the sampling in spring 2015 accounting for the same year, whereas the samples taken in autumn 2015 were counted for 2016. This led to an elongated period without testing dairy farms from autumn 2015 to autumn 2016. At the time this decision was taken, the prevailing opinion was that the virus had almost been eradicated, so surveillance could be considerably reduced. Unfortunately, this turned out not to be the case at all. The eradication scheme suffered a severe setback, with yearly case numbers doubling from 2015 to 2017 (Figure 2). As a consequence, epidemiological investigations were increased in autumn 2015 (compare chapter next chapter), and the surveillance was intensified in 2017 with testing of dairy herds again twice per year and in 2019, testing all other herds increased to once per year. Despite the outbreak in 2017 mainly affecting dairy herds, it is most likely that the sharp regional rise in cases in that year was linked to a cluster of heavily interconnected herds and individual non-compliance with the control programme. However, it is safe to assume that reduced surveillance certainly contributed to the steady increase in the spread of BVD in the years 2012–2017 (Figure 2). It was not until 2019 that another consequence of the 2017 outbreak became apparent: the seropositivity rate of bulk milk samples increased considerably, as seropositive replacement heifers from herds affected by the previous outbreak were often moved to other herds where they come into lactation. This led again to an increase in the number of positive bulk milk samples and consequently, the number of spot tests required, further increasing the costs and the workload for the regional veterinary services.

The regional veterinary services responsible for sampling on-site estimated the workload as being too high if all non-dairy herds were to be sampled yearly. To overcome this limitation, the project RiBeS (“Rinderbeprobung am Schlachthof”; sampling of cattle at the abattoir) was initiated in 2016 to take blood samples for surveillance in cattle during meat inspection at large abattoirs, as similarly proposed later in Japan (63). Sampling by RiBeS was simultaneously used for additional projects, e.g., related to bluetongue virus, bovine herpes virus-1, or enzootic bovine leukosis (bovine leukaemia virus). Thus, the frequency of monitoring non-dairy herds should be increased by RiBeS without the high workload arising by sampling on-site. But in contrast to the assumption that blood sampling at abattoirs would intensify monitoring of non-dairy farms, it turned out that the coverage of the population was actually lower. One reason for this decrease was clearly the fact that the project was still in its infancy and blood samples could only be taken at two of the eight large-scale slaughterhouses in 2016. This problem was solved, and sampling at the abattoirs reached the expected level in 2017 and even increased in 2018, enabling an increase in the surveillance of non-dairy herds to almost a yearly interval.

A spot test is considered positive if at least one animal is serological positive. For small herds, the size can be reduced from five to two animals as it was sometimes impossible to find more animals fulfilling all the requirements. In situations where only one animal is positive, the regional veterinary services perform a risk analysis to determine whether a suspected case is established and whether measures should be imposed on the herd. Spot tests in dairy herds are always taken from animals living on the farm by a veterinarian on a single day. Since 2018, spot tests from non-dairy herds are mostly taken during meat inspection at the abattoirs. The two big differences compared to the sampling in dairy herds by the classical spot test is that (i) the sampling takes place over a prolonged period and (ii) no second sample can be taken from the tested animals. Consequently, mistaken sample identification or false-positive test results are more difficult to verify. Evaluation of the results of the spot tests and the bulk milk samples indicated that in 2018, in about 89% of the positive screening results, no PI animal could be identified. Reasons might be that the animal might already has left the herd, or the spot tests were false-positive in both herd types. This provides evidence that serologically positive animals are still not restricted to animals that had contact with known PI animals. As a consequence, these seropositive animals are a major problem for effectively targeting the control efforts only to the herds where active transmission is indeed occurring.

### 2019ff: The Endgame?

As a result of these constantly high surveillance efforts, case numbers have dropped again from 258 infections in new herds in 2017 to 121 in 2020 (Figure 2). Experience gained in recent years clearly showed that (i) early reductions in surveillance and (ii) gaps in case investigations severely jeopardise the success of the eradication scheme. Concerning the former, surveillance in dairy herds continued with two samplings per year, whereas monitoring of non-dairy herds was increased considerably. This was achieved by programming an application for mobile phones (RiBeS-App) to identify bovines that should be sampled at an abattoir, which enables the collection of blood samples in almost all slaughterhouses in the country, including the smaller facilities. This led to a marked increase in the average percentage of non-dairy herds that were tested yearly by spot tests (Table 1). Nevertheless, especially in smaller cantons with no large slaughterhouse, only about a third of all samples required for the spot tests could be taken at the abattoirs, requiring more elaborate, and costly blood sampling on the farms. Overall, the number of serological tests conducted within the surveillance scheme has doubled to 65,000 from 2016 to 2018. In case a new PI animal is identified, detailed and timely contact tracing is required, investigating all possible exposures retro- and prospectively. Thus, the possibility that the same source of infection might have “laterally” generated additional PI animals in addition to the one detected by surveillance needs to be considered, as well as the possibility that the newly identified PI animal already led to the infection of other pregnant animals. The measures for the herds with positive animals remained about the same during the eradication programme, but the investigations for suspected cases, for example if a spot test was positive but no PI animal could be detected, were clearly intensified. With the help of computerised epidemiological tracing and targeted testing, PI animals were regularly detected earlier than would have been the case by the serological surveillance scheme. This is also apparent by the number of virological tests performed during these control measures that increased 3-fold from 2016 to 2018 to 30,000 analyses per year. This permitted the veterinary authorities to stabilise the situation, and case counts are decreasing since 2017.

**Table 1:** Bulk milk testing and fraction of non-dairy herds with complete spot tests per year in 2013–2020.

**Year**	**Bulk milk tests in dairy herds**					**Spot tests completed in non-dairy herds**
	**Total [*n*]**	**Yearly testings [*n*]**	**Negative [*n*]**	**Non-negative [*n*]**	**Non-negative [%]**	**[% of herds)**
2013	39,503	2 (S & A)	29,276	10,227	25.89%	33.8%
2014	42,539	2 (S & A)	37,494	5,045	11.86%	31.5%
2015	20,159	1 (S)	19,314	845	4.19%	20.2%
2016	19,478	1 (A-15)	18,217	1,261	6.47%	33.3%
2017	38,714	2 (A-16 & A)	37,977	737	1.9%	14.4%
2018	36,979	2 (S & A)	36,084	895	2.42%	23.3%
2019	36,198	2 (S & A)	34,275	1,923	5.31%	80.9%
2020	35,608	2 (S & A)	34,024	1,584	4.45%	n.a.

*The total number of bulk milk samples taken and the number of samplings per year from dairy farms that had to be sampled according to the eradication scheme are indicated. Samples were classified according to their ELISA PP-values as described in section Detection of Antibodies, with samples assigned to class 0 or 1 being regarded as negative, and to class 2 or 3 as non-negative. The percentage of non-dairy herds that were annually surveyed by spot tests are given, but do not represents a precise determination as no detailed data were available. In 2013–2018, one third of all non-dairy herds were to be tested by spot tests, whereas all of them should have been tested in 2019*.

*S, Spring; A, autumn; A-15, autumn 2015 counting for the year 2016; A-16, autumn 2016 counting for spring 2017); n.a., data not available*.

### Data Management Systems

The conceptual layout of the Swiss computerised data management has been previously described (54). In short, the centrepiece is the computerised information system (ISVet) of the Swiss Veterinary Service, which provides automated documents for both, the Veterinary Service and private veterinarians, on all aspects relevant to veterinary public health. Specific data and documents are accessible by different user groups, (i) via a BVD-Web platform for practitioners, (ii) via ISVet for the Veterinary Services and (iii) the Swiss animal movement database (AMD) for farmers. Results from all laboratory tests for BVD are transmitted to a centralised laboratory database run by the Federal Food Safety and Veterinary Office (FSVO), which is itself connected to the data on the herds and the animals in ISVet. As IT systems are generally not long-lived and given the long duration of this control programme that began in 2008, a new laboratory information system database (Alis) containing a more detailed data structure was introduced in 2013. Similarly, ISVet was replaced by “Asan,” but given the complexity of the ongoing BVD control programme in ISVet and the expected costs of transferring the whole functionality to the new application, it was decided that ISVet should remain functional exclusively for the BVD control programme. As a downside, the routine in using ISVet was lost when experienced users need to be replaced by new operators accustomed to Asan, and technical support for ISVet was greatly reduced in the belief that it would be shut down completely after a few years—which was obviously not the case.

The applications RiBeS and RiBeS-App, used to indicate which animals the meat inspectors need to sample at the abattoirs, are completely independent from the other software applications, but they provide an interface with the resource-planning software of the enterprise of the large and small abattoirs, respectively. Taking samples according to RiBeS is now well-established in the meat inspection process. Nevertheless, food safety clearly remains the priority in the process of meat inspection, and the additional effort needs to be financially compensated. To profit from possible synergisms, a more integrated system from management to integration of laboratory results for all cattle surveillance programmes is planned in the future. A more detailed data structure would be of great value especially for the spot tests, as with the current systems, differentiation of the results of the spot test from the ones of other serological assays in dairy herds proved to be difficult.

In the last 3 years, the use of a data warehouse combining information from different sources allowed the production of useful reports for epidemiological investigations and contact tracing of animals and herds with positive results in serological surveillance. These possibilities are increasingly used by the regional veterinary services. The format of flexible reports combining the information from the AMD, the laboratory database, ISVet, and RiBeS proved to be an important improvement. The animal movement database is the most important source for tracing of animals, and current efforts are directed toward specifically transforming data available from the AMD into information useful for BVD control, such as the calculation of calving periods and the proportion of twins and stillbirths per herd. The data management systems used offer great flexibility, but as a disadvantage, retrospective evaluations are rather difficult as the previous status of animals and herds are not available, e.g., in contrast to data management systems used in Germany (64).

## Virus Transmission

As virus is shed from all secretions, e.g., saliva, semen, tears, milk, and to a lesser extent faeces, direct contact of susceptible animals to persistently or transiently infected cattle is the most prominent way of horizontal transmission. Summer grazing on one of the 6,740 communal alpine pastures (as of 2019) is very common in Switzerland (65), with approximately one third of all cattle being moved to these pastures every year (55). This offers ample opportunity for direct contact of animals from different farms and, therefore, for the virus being transferred to different premises (66–72). As the virus can retain its infectivity for several hours or even days depending on the environmental conditions (73), spread by indirect contact through contaminated surfaces, fomites, equipment, vehicles, personnel, and even veterinarians cannot be excluded. Contaminated biological products such as semen or vaccines and even airborne transmission were reported to be possible routes of transmission [(49, 74, 75) and references therein]. As examples, BVDV transmission was reported from external contamination of rubber membranes of vaccine vials that were punctured by the syringe (76), from orf vaccines for sheep that were contaminated with BVDV-2 (77), from contaminated transport vehicles (78, 79), or by airborne transmission via short distances of maximally 10 m from pens harbouring a PI animal (76, 80).

### Transmission From PI and TI Animals

As PI animals constantly shed large amounts of viruses during their whole lifetime, transmission from these animals is highly effective. This is exemplified by the facts that a within-herd seroprevalence of at least 60–70% is highly indicative for the presence of a PI animal (60), and that the presence of a PI calf for only 1 h was sufficient to infect the contact animals (81). Thus, the basic reproductive number (R_0_), which indicates the expected number of new infections generated by one case in a completely susceptible herd, might be well above 30 for the transmission by PI animals (75). This is in accordance with the model that PI animals are the most important reservoir for BVDV to remain in the population.

Viremia in transiently infected (TI) animals starts at around 2–3 days post-infection (p.i.), and last up to 1–2 weeks until seroconversion of the infected host occurs. This indicates that the presence of infectious virus in secretions from acutely infected animals can be expected. Indeed, infectious virus could be isolated from nasal swabs from 5 out of 6 experimentally infected animals between day 5 and 10 p.i. (82), whereas this could be extended up to 21 days p.i. by treatment of the animals with dexamethasone (83). Similarly, 10 calves infected intranasally all seroconverted within 15-36 days p.i., and BVDV could be isolated from some of these animals between day 5 and 8 (84). In another study, animals were acutely infected by contact to a PI animal, but no infectious virus could be isolated from nasal swabs of these contact animals despite positive detection of viral RNA by RT-PCR in blood and nasal swabs starting at 6–21 days p.i. and lasting for 1–9 days (85). It is worth noting that detection of virus in serum or nasal swabs by RT-PCR depends on the dose of virus used to infect the animals (83), and can be detected up to ~100 days p.i. despite interim seroconversion (83, 86). However, virus isolation in cell culture indicative of the presence of infectious virus was not successful at the late time points. Interestingly, blood transfusion with blood at day 98 p.i. from acutely infected animals to naïve cattle led to seroconversion of the latter, indicating that virus in the blood still retains infectivity despite being unable to be transmitted naturally to sentinel animals (86).

This rather short time window of virus secretion together with a reduced amount of virus shed by acutely infected compared to PI animals leads to strongly reduced efficiency of virus transmission. Thus, none of the 14 sentinel animals were infected by nose-to-nose contact with 5 TI calves (87). This was confirmed in another study by the same group where 8 calves exposed to 10 TI animals were not infected despite the detection of BVDV in nasal swabs in 6 out of 10 of the TI animals, whereas a bovine coronavirus was readily transmitted to all the animals (84). The infectious dose leading to a transient infection (83), the virulence of the virus strain involved (32), or concomitant infections, e.g., within the bovine respiratory disease complex (74), might further influence the efficiency of virus transmission from TI cattle, but data are rather scarce. A summary of studies that investigated transmission from TI animals is collected in Supplementary Table 2.

### Transmission via Semen

In rare cases, the persistence of BVDV in testicles of postpubertal bulls was described despite these bulls seroconverting after transient infection and being free of virus in serum thereafter. In these animals, infectious virus could be detected in semen for months (88–91), even though the viral load in semen from TI animals were considerably lower than in semen from PI bulls (92). Thus, pestivirus transmission by artificial insemination with semen from TI bulls could be observed, but secondary transmission cycles were only rarely described (88). Similar results were reported using semen from PI bulls, but despite high rate of seroconversion of the inseminated heifers (93–95), no (95) or only two PI animals (93) were generated out of 5 and 61 inseminated heifers, respectively. Therefore, transmission of ruminant pestiviruses via semen does occur, but the rate of production of PI calves and even less, further transmission to naïve animals, is remarkably low.

### Risk Assessment for Transmission From TI Animals

As BVD eradication in cattle was not achieved as quickly as expected and the source of infection could not be identified in several cases, doubts were raised that the focus on PI animals as the main source of infection could not be justified. Anecdotal accounts of transmission that appeared to have occurred through TI animals raised concerns that this route of transmission might be more common and jeopardise the control programme, and a risk assessment was appreciated by the local authorities and veterinarians. Overall, direct transmission from PI animals to naïve cattle remains the most prominent way of spreading ruminant pestiviruses (96, 97). As they shed virus throughout their life, PI animals of any age are effective transmitters (a few examples are summarised in Supplementary Table 3), with the possible exception of temporarily reduced viral shedding after intake of colostrum containing maternal neutralising antibodies (74, 98). There appears to be a consensus that the risk of transmission by TI animals is negligible and mostly unable to sustain a chain of infection for an extended time period (49). Indirectly, this is corroborated as all BVDV eradication programmes were successful provided they aimed at the elimination of PI animals (45). Calculations of the reproductive number R_0_ were rarely done, and the results were quite diverse, but in most cases, R_0_ for TI animals was below 1. Thus, R_0_ was reported to be around 0.25 for BVDV-1 and−2 being transmitted by experimentally generated TI animals, whereas the introduction of PI animal led to an unlimited increase of R_0_ [“R_0_ = ∞” (96)]. In accordance with this very high R_0_ in the presence of a PI animal, herd immunity would need to be close to 100% to achieve full protection, which is not realistic (99). By contrast, a previous study done in the Netherlands reported an R_0_ of ~3.3 in a herd that did not contain a PI animal (30). However, PI animals were at least temporarily on the premise in different pens, and the chain of infection ceased before infection of all naïve cattle, indicating that R_0_ might nevertheless have been below 1 for transient transmissions only. Surprisingly, the within-herd transmission was rather slow in the presence of a PI animal, with an R_0_ of only 3.9 reported in his study (30). In a mathematical model, R_0_ was calculated to be 2.3 in the absence of a PI animal but was increased by an order of magnitude by the introduction of a PI calf (100).

Summarising these studies (Supplementary Table 2), transmission from TI cattle to contact animals at physiological conditions occurred in only 3 out of 60 cases, with additional transmission only in the case of immunosuppressed calves (83). Out of this, an R_0_ of 0.05 (95% CI; 0.01–0.14) can be estimated. Of course, herd size, cattle management, general health status etc. will influence the efficiency of transmission, but TI animals appear to be an even smaller risk than surface or fomite contamination by secretions of PI animals, especially during the birth of PI animals. Therefore, the detection of TI animals is not the main risk factor to maintain a chain of infection, but rather represents an important indicator of the presence of a source of infection, e.g., a PI calf. In later stages of BVDV eradication with a highly susceptible cattle population and intense surveillance, transient infections might nevertheless be observed and might lead to either costly investigations or, in rare cases, to the transmission to a pregnant heifer and the birth of new PI calf.

## Diagnostics

Since the first description of BVDV in 1946 (101, 102), a number of methods were developed to directly identify the virus or its components, and indirectly to monitor seroconversions as signs of infection. Diagnostic tests are applied either to diagnose clinical cases, or to survey groups of animals to determine the (sero-) prevalence of infection. In the case of a BVD eradication programme, the latter clearly applies, as most acute and persistent infections are inapparent, and the ultimate goal is to identify every PI individual. The special biology of ruminant pestiviruses as described in the first chapter, with acute infections characterised by transient viremia followed by seroconversion, and the presence of immunotolerant PI animals, requires the application of various diagnostic assays and detailed interpretation of their results. In addition, possible interference by maternal antibodies imposes the selection of different tests depending on the age of and the type of sample taken from the animal. A large body of literature is available on diagnostics tests, but in the following paragraph, we concentrate on the assays used in Switzerland during the BVD eradication scheme and discuss pitfalls observed in this “large field experiment”. Thus, we apologise that we are only able to cite a small number of articles which by no means detract from the effort made by many labs to improve diagnostics of this important livestock disease.

### Detection of Antibodies

For detection of antibodies, serum neutralisation test (SNT) is highly sensitive, and was and still is the gold standard, but the requirement of cell cultures limits its use to more specialised laboratories. Thus, agar gel immunodiffusion test and enzyme-linked immunosorbent assay (ELISA) were rather routinely applied (103, 104), with the former not being used in Switzerland compared to, e.g., Australia or New Zealand (105). Today, a number of indirect and blocking ELISAs to detect antibodies to ruminant pestiviruses are commercially available that can be used with various sample materials such as serum, plasma, or milk. Most of these ELISA tests use the non-structural protein NS3 (p80) as capture antigen as this is the most conserved pestivirus antigen, with fewer assays detecting antibodies to the structural protein E^rns^ (106, 107). By contrast, neutralising antibodies are primarily directed against the envelope glycoprotein E2, which at least partially explains discordant results that were reported between antibody ELISA and SNT (108).

In countries where HoBi-like pestiviruses (Pestivirus H) are circulating, specific assays need to be developed as the test routinely applied for BVDV and BDV appear to unreliably detect these types of antibodies (109–112). Independent of the type of ELISA used, none are currently able to differentiate BVDV antibodies from BDV. Correctly attributing antibodies to one of the species requires cross-neutralisation assays using different virus strains as challenge virus. This type of assay needs to be adjusted to the corresponding epidemiological situation, i.e., to the individual types of viruses circulating in a given area. Currently, using two strains of BVDV-1, one that is and one that is not circulating in Switzerland, and one local BDV strain, enables at least 80% of all sera to be designated to one of the two ruminant pestiviruses [(108), and Huser et al., in revision]. Requiring cell cultures and three separate SNTs for cross-neutralisation, this test is rather elaborate, time consuming and costly and, thus, is performed exclusively by our reference laboratory and only upon request of the corresponding veterinary authority.

Detection of new antibody-positive animals or a rise in the level of antibodies in bulk milk during the surveillance phase is indicative of the presence of a PI animal in a herd. As a result, investigation at the farm level with analysis of every individual animal in the herd is required. Cows shortly around calving are also tested in such cases, yielding sometimes negative results in antibody ELISA despite records indicating that the animal was previously tested antibody positive. This might be explained by the fact that cows around parturition actively transfer enormous amounts of antibodies of the IgG1 subtype from serum into the mammary gland, thereby assuring colostrum-mediated protection of the newborns. Depending on the antibody ELISA used, this drop in antibody levels in the serum of the cow might lead to a negative result around parturition (113, 114) and, therefore, it is not recommended to perform antibody ELISAs ~2 weeks before and after calving. This effect is not specific to BVDV antibodies, as IgG1 antibodies in general are transported into the milk as, amongst others, was reported for antibodies to *Coxiella burnetii* in addition to BVDV (113, 114).

Every laboratory that offers BVDV diagnostics in Switzerland needs to be accredited, and every test applied in these laboratories requires approval by the federal authorities. Switzerland is a federalistic country and, therefore, the implementation of the national eradication programme is organised by the 23 different cantons, with possible collaborations between some of the cantonal veterinary services. Accordingly, every canton is free to choose a laboratory for its analysis, and each accredited laboratory is free to choose which test to use as long as the test was approved. All these ELISA tests were reported to have sensitivities and specificities above 90% using serum as sample material (106, 115–118), with somewhat lower values using milk samples (117). Despite these similar characteristics for the different tests, it appeared that the performance varied between different regions using various ELISAs. This was also recently confirmed where various commercially available ELISA tests generated false negative results, especially in samples with low antibody titres according to a neutralisation test (119). However, it must be kept in mind that it is not only the test that is responsible for inaccurate results. Correct labelling of the sample, quality of the sample material, duration of and temperature during shipment and correct handling during analysis all contribute to the final result. In such a field situation, no test can be 100% accurate, and the occurrence of false-positive or negative results cannot be completely avoided. To reduce this variability, the analysis for antibodies in bulk milk during the surveillance phase in recent years, i.e., toward the end of the eradication programme, is performed by a single laboratory for the whole country. Bulk milk samples are collected twice monthly for quality control of commercial milk (milk testing) from all dairy herds, and such samples are used twice a year for BVD monitoring during a defined collection period. The bulk milk samples are analysed using the SVANOVIR® BVDV-Ab ELISA from Svanova (now Indical Bioscience). Based on their PP values (percentage positivity value), farms are assigned to one of four classes defined by the test manufacturer according to the Swedish national programme (120), i.e., class 0 (PP < 3%), class 1 (PP ≥ 3% and < 14%), class 2 (PP ≥ 14% and < 30%), and class 3 (PP ≥ 30%). Nevertheless, analysing the sample by one laboratory only does not eliminate all pitfalls, as with the analysis of bulk milk samples in spring and fall each year, an inexplicable rise in antibody titre could be observed in some farms, without detection of a PI animal following investigation of all animals in the herd (unpublished observation). At least in some cases, the purchase of an antibody-positive cow could be identified as the cause of the rise in bulk milk antibodies, or the new animal was even the only seropositive animal in the herd, with the bulk milk antibody level returning to background level after drying off of this seropositive cow. Thus, a single animal with a high antibody level in milk can unfavourably influence antibody surveillance by bulk milk analysis.

Currently, every blood sample that tested positive or indeterminate by an external laboratory must be transferred to our reference laboratory for confirmation. If the sample yields discordant results with an indirect and, if necessary, an additional competitive antibody ELISA, the final analysis will be done using SNT. If no neutralising antibodies are detected in the sample, the result will be reported as negative. Expensive and time-consuming cross-SNT is currently the only way to differentiate antibodies from BVDV to BDV, and is solely performed by the reference laboratory. In addition, inappropriately re-sampling animals only a few days after the initial test in response to farmer's request for a definitive result, and testing animals in the periparturient period, are additional drawbacks regularly encountered with antibody testing.

### Detection of Viral Antigen and Viral RNA

For the direct detection of virus, virus isolation has been considered the gold standard for many decades and is the only test able to detect infectious virus (104, 121). Similar to the SNT for the detection of antibodies, it is time consuming and costly, and it requires laboratories capable of performing cell cultures. In addition, the method is sensitive to the presence of antibodies, e.g., maternal antibodies from colostrum intake in young calves (122, 123), and the sensitivity is highly dependent on the cell type used, with bovine turbinate cells being up to two orders of magnitudes more sensitive to infection by BVDV than the commonly used MDBK cell line [(115) and unpublished observation]. But as “all that glitters is not gold,” it is rather RT-PCR than virus isolation that is nowadays accepted as the most sensitive assay (see below). For the detection of viral antigens, immunofluorescence and immunohistochemistry was initially the method of choice, but antigen ELISAs replaced these assays in routine diagnostics (104, 106, 115, 124–126). The non-structural protein NS3 was the most common antigen detected by these ELISAs, yielding the most sensitive results when using buffy coat as sample material. Accordingly, flow cytometry was used in some specialised laboratories to detect intracellular NS3, which also enabled the identification of the cell type infected (127, 128), but this was never routinely applied in Switzerland. In addition to NS3, ELISAs detecting E^rns^ in serum were commercialised, which enables the use of serum to detect the soluble form of this envelope glycoprotein (129, 130). Similar to virus isolation, the antigen ELISA might yield false-negative results due to the presence of maternal antibodies (70, 122, 123). With a half-life of ~20–30 days, passively acquired antibodies largely wane within two to four months (122, 131). Interestingly, maternal antibodies directed against the viral envelope glycoproteins E^rns^ and E2 decline at a faster rate in PI animals compared to naïve calves, whereas antibodies to NS3 wane at around equal rates in PI and non-infected animals [(122) and unpublished observation]. This probably reflects the presence or not of the corresponding antigen in the serum. Including a certain “safety margin”, antigen ELISA is therefore only used in animals older than 6 months.

These days, RT-PCR and real-time RT-PCR detecting viral RNA are the methods of choice next to the antigen ELISA that are routinely used to demonstrate an infection with pestiviruses (107, 118, 132–134). A wide variety of sample material can be used, such as blood, saliva, ear notches, milk, or different material from abortions, provided appropriate methods for RNA isolation are established for each of the sample types. It is worth mentioning that material from the afterbirth might test negative by RT-PCR despite the birth of a PI animal after transient infections of its dam, which might relate to the fact that the foetal rather than the maternal side of the placenta is virus positive (135), albeit more studies are required to confirm this observation. For diagnostic purposes, the 5′-untranslated region (5′-UTR) is usually chosen as PCR target as it is the most conserved region in pestiviruses, enabling the simultaneous detection of various genotypes. Accordingly, the—in the meantime famous—“Vilcek pan-pesti primers” are sometimes still in use (136), albeit adapted primers were designed covering the many new pestivirus isolates identified in recent time. Nevertheless, all the varying pestiviruses cannot be detected using a single PCR and depending on the epidemiological situation and the species to be investigated, specific primer/probes need to be designed. In Switzerland, only BVDV-1 and BDV were identified in livestock and wild ruminants to date by using a broadly specific RT-PCR for sequencing using a mix of different forward primers (79) that enables the detection of a variety of pestiviruses, e.g., pestiviruses, A, B, D, and H.

**Figure 3:**
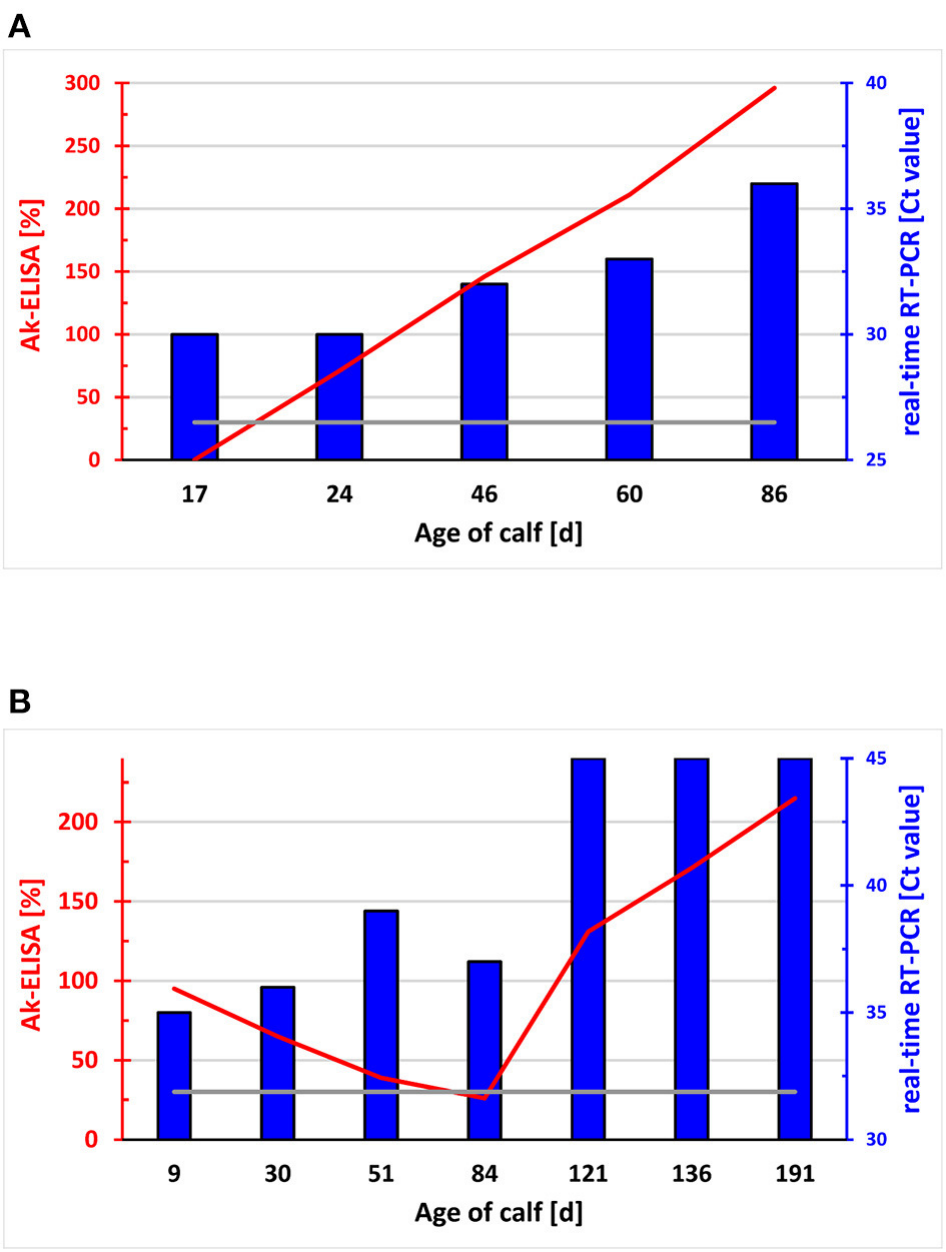
Course of the level of pestivirus antibodies and viral RNA in the blood of newborn calves. Relative optical density (OD) in the ELISA for pestivirus antibody in sera of two calves taken between day 17 and 86 **(A)** and day 9 and 191 **(B)** of age expressed as a percentage to the OD of a standard serum (y-axes to the left in red). Relative OD values > 30% (grey line) are defined as positive. The presence of viral RNA by real-time RT-PCR, and the Ct values are indicated (y-axes to the right in blue). The calf in **(A)** did not receive pestivirus antibody-containing colostrum, whereas the calf in **(B)** ingested maternal antibodies to BDV. The virus detected by RT-PCR could be identified as BVDV-1b [**A**; (79)] and BDswiss [**B**; (145)], respectively.

As RT-PCR is largely unaffected by the presence of maternal antibodies, it is always used with samples from animals younger than 6 months of age. Due to its high sensitivity, RT-PCR allows pooling of samples (137, 138) followed by re-analysis of individual samples exclusively from positive pools. With a prevalence of PI animals of roughly 1-2%, this considerably reduces the costs of large-scale investigations. Based on the lower sensitivity, it is not recommended to use the antigen ELISA test with pooled samples (107, 139). In addition, the high sensitivity of RT-PCR not only allows efficient detection of PI animals, but transient infections might also provide a positive result when the animal was sampled during the viremic phase. In general, the viral load in PI calves is higher than during the short viremia found in TI animals, resulting in lower C_t_ values in real-time RT-PCR in PI compared to TI animals. Despite this difference being highly significant on a population level (140), it cannot be applied to differentiate PI from TI animals in individual cases. Thus, the C_t_ values vary widely, as we observed samples from PI animals providing Ct values above 35 or TI animals showing C_t_ values lower than 20 [(33) and unpublished observation]. Re-sampling the animal at least 3 weeks later resolves the issue in most cases, as the viremic phase in TI animals is usually rather short-lived. However, infections of neonates or very young calves with BVDV might result in a prolongation of viremia due to either an inefficient immune response in young animals (Figure 3A) or by the inhibition of the calf's immune response by immune complexes of the virus with antibodies obtained by colostrum intake (Figure 3B). In the latter case, even immunohistochemical staining in ear notch biopsies stained positive for pestiviral antigen (unpublished observation), despite this method being supposed to exclusively detect PI animals (141). Thus, the results of RT-PCR assays of a single test and even together with the results of a paired sample taken at a later time point, needs to be interpreted in relation to the epidemiological context of the animal, results from other animals or the seroprevalence in the herd, in order to obtain a definitive conclusion and to take the appropriate measures. Finally, it has to be noted that it is not possible to identify PI animals already *in utero*, at least not with routine methods (142). Pregnant cattle carrying a PI foetus (metaphorically called “Trojan cows”) mount a strong humoral immune response, with neutralising titres being much higher than in transiently infected naïve animals (143, 144). This difference, however, is only significant in the last 1–2 months of pregnancy and is not commonly amenable for diagnostic purposes at the level of individual animals. Consequently, intake of maternal antibodies by the PI animal taking colostrum from its own mother is substantial and one should be aware of their possible interference with the various diagnostic tests as discussed above.

In summary, antigen ELISA and real-time RT-PCR are the methods routinely used in Switzerland during the BVD eradication programme, and both tests exhibit an excellent performance. However, no test can perform at 100% sensitivity and specificity in field situations, a fact that tends to be neglected by a number of stakeholders. As only BVDV-1 and BDV strains are circulating in domestic and wild ruminants in Switzerland (34, 79, 146, 147), the pestivirus diversity is currently not an issue for the diagnostic methods used to detect the presence of pestiviruses. Nevertheless, BVDV-2 or HoBi-like pestiviruses, which probably represent the greatest risk of being introduced into the Swiss cattle population, can be identified by the methods currently applied. The major pitfalls in antigen detection are (i) that, especially in young calves, viremia might persist for several weeks to months at a low level, making differentiation of TI and PI animals rather difficult, and (ii) that re-sampling of initially positive animals occurs too fast, sometimes within 1 week, which makes it more or less impossible to follow the course of infection.

## Small and Wild Ruminants

Ruminant pestiviruses are not strictly species specific and thus, infection from small ruminants such as sheep and goats were described in the field as well as under experimental conditions. The presence of BDV in cattle was already discussed in a recent review (145) and, therefore, only aspects relevant for BVD eradication are covered here. Commingling of cattle with persistently infected sheep led to seroconversion, reduced fertility and abortions in pregnant animals (148–150). The declining seroprevalence during the eradication leads to a completely susceptible Swiss cattle population, and there were concerns that the generation of cattle PI with BDV will strongly increase. However, within almost 10,000 nucleotide sequences obtained from virus isolates taken from PI (and possibly some TI) animals since the start of the eradication programme, not a single case of BVDV-2 and <30 animals PI with BDV were identified [(79); Huser et al., in revision]. Interestingly, most of these PI animals were detected in Central and Eastern Switzerland, probably reflecting different management practises of keeping cattle and sheep on the same premises or pastures in various regions in Switzerland, including communal alpine pastures in summer (67, 68, 70, 72). This is corroborated by the observation that cases of malignant catarrhal fever in cattle, a disease caused by ovine herpesvirus-2 with sheep representing symptomless carriers, are similarly concentrated in Central and Eastern Switzerland (Huser et al., in revision).

Due to cross-reactivity of antibodies to pestiviruses, serological surveillance of BVD by ELISA does not distinguish between BVD- and BD-virus as the source of infection. In a recent study using an optimised SNT protocol, we could show that <10% of pestivirus antibody ELISA-positive sera from cattle were due to BDV infection (108). The samples were taken between 2012 and 2014, and there was a trend for an increased BDV seroprevalence in these samples from 4.2 to 8.1%, which might reflect the increased susceptibility of the cattle population. Epidemiological analysis revealed that common housing of cattle and small ruminants, especially sheep, was the most significant risk factor for BDV infection in cattle. Goats appear to be less of an issue as PI goats appear to be rarely generated and their viability is mostly severely reduced (151). As observed for the presence of cattle PI with BDV, the highest BDV-seroprevalence in cattle was found in Central Switzerland.

These data indicate that sheep might represent a reservoir for ruminant pestiviruses, but their transmission to cattle occurs only sporadically and largely depends on herd management. Direct contact between these two species represents the highest risk for transmission but contact between cattle and sheep on neighbouring pastures and insufficiently cleaned trailers commonly used by a cattle and sheep farmer could be identified as sources of infection (79). However, as routine antibody surveillance by ELISA does not discriminate between antibodies to BVDV and BDV, the suspicion of BDV in sheep being the source of infection can only be raised based on indirect evidence. Thus, the following observations were reported that might indicate that BDV was introduced into a farm: (i) seropositive results in bulk milk or in the spot test of young calves that are inexplicable as no PI animal could be found in the herd; (ii) possible direct or indirect contact to small ruminants; (iii) only a few seropositive animals could be identified; (iv) the values in the antibody ELISA of the sera of seropositive animals are only weakly positive; or (v) the ELISA results of bulk milk analysis was low or even negative despite the presence of lactating seropositive animals in the herd. The latter two observations might be explained by the fact that the ELISA OD (optical density) values of BDV antibodies appear to be generally lower than the ones measured by BVDV antibodies (unpublished observation). But even in case where BDV is suspected to have been introduced into a cattle herd, the same enforcements as applied for BVDV should be immediately taken, as in-depth analysis to differentiate the pestiviruses takes time, whereas further transmission should be stopped as quickly as possible.

Previous studies showed that the pestivirus seroprevalence in sheep was around 15–20% (152–154). Identification of the type of pestivirus infection in sheep, if determined at all, showed a considerable proportion of BVDV-induced antibodies, albeit 30–60% of the samples could not be allocated at that time. Hence, it could be envisaged that the elimination of BVDV from the cattle population would decrease the transfer of BVDV from cattle to sheep and thereby altering the epidemiology of pestiviruses in small ruminants. Indeed, analysing sheep sera collected in the Canton of Schwyz in Central Switzerland ~7–10 years prior to and after the start of the BVD eradication in cattle revealed that the proportion of antibodies to BVDV compared to BDV decreased from 13.3 to 3.5% between the early and late sampling period (Huser et al., in revision). This provides strong evidence that there is not only cross-species transmission of BDV from sheep to cattle, but also significant transmission of BVDV from cattle to sheep and, therefore, BVD eradication in cattle is also of benefit for the sheep, despite BDV remaining endemic in the sheep population.

In addition to small ruminants, a number of wild animals were found to have been infected by ruminant pestiviruses [for reviews, see (155–158)]. However, evidence for independent virus circulation within the wild animal population without the involvement of livestock was rarely found with possible exceptions in chamois in the Pyrenees in France and Spain and white-tailed deer in North America (159, 160). In Switzerland, roe deer, red deer, chamois or ibex were considered to be virus reservoir for pestiviruses, thereby representing a potential risk factor for BVDV eradication in cattle. However, none of the roe deer analysed, and only, 2.7, 2.1, and 1.8% of red deer, chamois, and ibex, respectively, were seropositive (146) out of a total of 1,877 samples analysed. Differentiation of approximately half of the seropositive samples indicated that the majority of wild ruminant sera contained antibody to BDV rather than BVDV (147). This might be corroborated by the observations that using RT-PCR, only one single serum from a chamois contained viral RNA that could be typed as BVDV-1 h (146), the most prominent genotype found in cattle in Switzerland (34). These data indicate that wild ruminants in Switzerland do not represent a pestivirus reservoir but are rather an incidentally spill-over host and, therefore, do not pose a risk to BVD eradication in cattle. A similar conclusion was made when looking at wild and domestic ruminants in Southern Spain (161).

Overall, these data strongly suggest that small and wild ruminants in Switzerland are not a significant risk factor for BVD eradication in cattle. However, occasional spill-over transmission might occur from cattle to small and wild ruminants and vice versa, the latter mostly during alpine farming in summer. As the surveillance programme is based on the seronegativity of cattle herds, every transmission event detected during transmission requires further investigations to elicit the possible source of infection. Legally, this implies that infection of cattle with other ruminant pestiviruses such BDV (Pestivirus D) or HoBi-like viruses (Pestivirus H, never observed in Switzerland and, thus, not further discussed here), are not specified by the animal disease regulation (animal disease ordinance; “Tierseuchenverordnung TSV”). As routinely applied diagnostic tests do not differentiate between BVDV and BDV, any positive result is defined as a positive case with all its consequences defined in the TSV for BVDV. However, if antibody monitoring or further investigations on virus-positive cattle indicate that, e.g., a PI animal is infected with BDV, the TSV does not apply, and any further actions rely only on a general act regulating animal disease control measures in the animal disease law (“Tierseuchengesetz TSG”). In light of new pestiviruses that were recently described and might be discovered in future, a broader definition for pestivirus infection in cattle might be advantageous and legally assured in an animal disease regulation in countries with scheduled or ongoing eradication programmes. Similar problems were faced with the eradication of caprine arthritis encephalitis virus (CAEV) in Swiss goats, where sheep infected with Visna-Maedi virus (VMV) or CAEV play a significant role as a reservoir for such small ruminant lentiviruses (SRLV) and, therefore, as a risk factor in the control of CAEV in the Swiss goat population (162–166).

## Molecular Epidemiology

As stated above (chapter Data management systems), the enormous logistical tasks encountered in the eradication programme could not have been achieved without appropriate data management systems, including the animal movement database (AMD) (54). In the AMD, every single bovine animal with its unique ear tag number can be identified, incl. information such as date and place of birth, additional farm-related data, information on its animal parents, animal movement, slaughter or death, etc. As with any database, inaccurate or missing entries (167), either by negligence or fraudulence, should be avoided, as this might severely limit the practicality of the database. In addition to its role in the logistics of the testing scheme, this digital data system is also an invaluable tool used for contact tracing, an important instrument in classical epidemiology to identify a possible source of infection and further contact animals. In addition to the “classical” tools, molecular epidemiology is nowadays an important method in disease control (168–170). Accordingly, molecular epidemiology was successfully used in pestivirus control and surveillance, e.g., for CSFV (171–173) and various BVDV control schemes ins Scandinavia (45, 56, 174), the UK (175), Austria (78), Germany (176, 177) and Scotland (178). In Switzerland, we sequenced a short stretch of ~240 bp of the BVDV genome in the 5′-UTR from a large number of PI animals and combined this information with data from the AMD (34, 79). Initially, this sequencing effort intended to identify animals PI with BDV, but it was soon realised that these sequences are a great opportunity to be used in molecular epidemiology. On the one hand, we could gain an overview on the BVD viral strains circulating in Switzerland, which is important to control for possible introductions of new variants into the country and to monitor the suitability of current diagnostics tools. Next to a few PI animals infected with BDV as discussed above, we exclusively found BVDV-1 strains of the subgenotype BVDV-1b, −1e, 1h, and−1k with the exception of two isolates of the 1g and 1l subgenotype (34). Notably, we never found an animal PI with BVDV-2 or HoBi-like pestiviruses, despite these genotypes being described in neighbouring countries, i.e., BVDV-II in France, Germany, and Italy (177, 179–181), and HoBi-like viruses in Italy (182). On the other hand, we were able to support the cantonal authorities in tracking chains of infection, e.g., whether several PI animals in a single farm or on a single pasture originated from one or more virus introductions, or whether repeated births of PI animals on the same farm were caused by consecutive infections over time originating from the same source of infection or represented new virus introductions (79).

The very strict and ambitious BVDV eradication in Switzerland led to a quick initial success [Figure 2; (59)]. Since then, the eradication remains, however, somewhat in a stalemate. This notwithstanding, the good news prevails with more than 99.5% of all cattle farms being free of BVDV at the beginning of 2021. To obtain complete freedom from BVDV, it is of upmost importance to identify and remove the remaining PI animals as quickly as possible. This might be exemplified by the observations that a change in personal within cantonal authorities might have led to a temporal surge in the number of PI animals produced, as case investigations could not be followed in time with the rigour required. Similarly, the fact that the oldest PI animals from which we received a blood sample for sequencing in the surveillance phase was between 1 and 3.5 years of age—with an outlier in 2015 at an age of 7.3 years (Table 2)—indicates that a number of PI animals were clearly identified too late, giving them enough time to further transmit the virus, possibly also to naïve pregnant animals. Out of ~10,000 animals imported annually, only a handful of these adult animals were tested positive, and a few sequences might have been obtained from TI animals, indicating that the majority of the sample sequences were indeed from PI animals infected in Switzerland. Thus, some of these older animals must either have been missed in the surveillance scheme, or they were previously tested false negative. And these are not only single cases that were detected exceptionally late, but around 10% of all samples from PI animals we received for sequencing were from animals of 6 months of age or older (Table 2).

**Table 2:** Age of the PI animal when it was sampled and sent to the reference laboratory for sequencing.

**Year**	***n***	**Min [d]**	**Max [d]**	**Average [d]**	**Median [d]**	**> 180 d**	**> 180 d [%]**
2008	4,001	10	3,525	378.4	236	2,258	56.4%
2009	1,945	3	3,282	79.3	26	126	6.5%
2010	958	0	1,570	80.6	20	63	6.6%
2011	460	1	1,502	66.6	16	21	4.6%
2012	108	3	934	40.7	18	3	2.8%
2013	75	5	1,285	77.8	18	6	8.0%
2014	98	2	1,112	100.3	26	15	15.3%
2015	219	1	2,661	100.1	18	25	11.4%
2016	295	0	952	61.2	14	26	8.8%
2017	473	1	1,245	90.3	33	71	15.0%
2018	321	1	609	81.8	32	40	12.5%
2019	252	1	970	61.4	12	22	8.7%
2020	203	2	1,039	81.0	13	25	12.3%
2021^*^	32	7	368	67.4	44	3	9.4%

*The year of sampling and the number of animals analysed is indicated, with the age (always given in days [d]) of the youngest (min) and the oldest (max) animal in addition to the mean and medium ages of all animals tested per year is provided. The number of animals that were older than 6 months at the time of sampling are given in absolute [d] and relative [%] numbers per year of sampling*.

* *Data collected by the reference laboratory until Feb 9th, 2021*.

**Figure 4:**
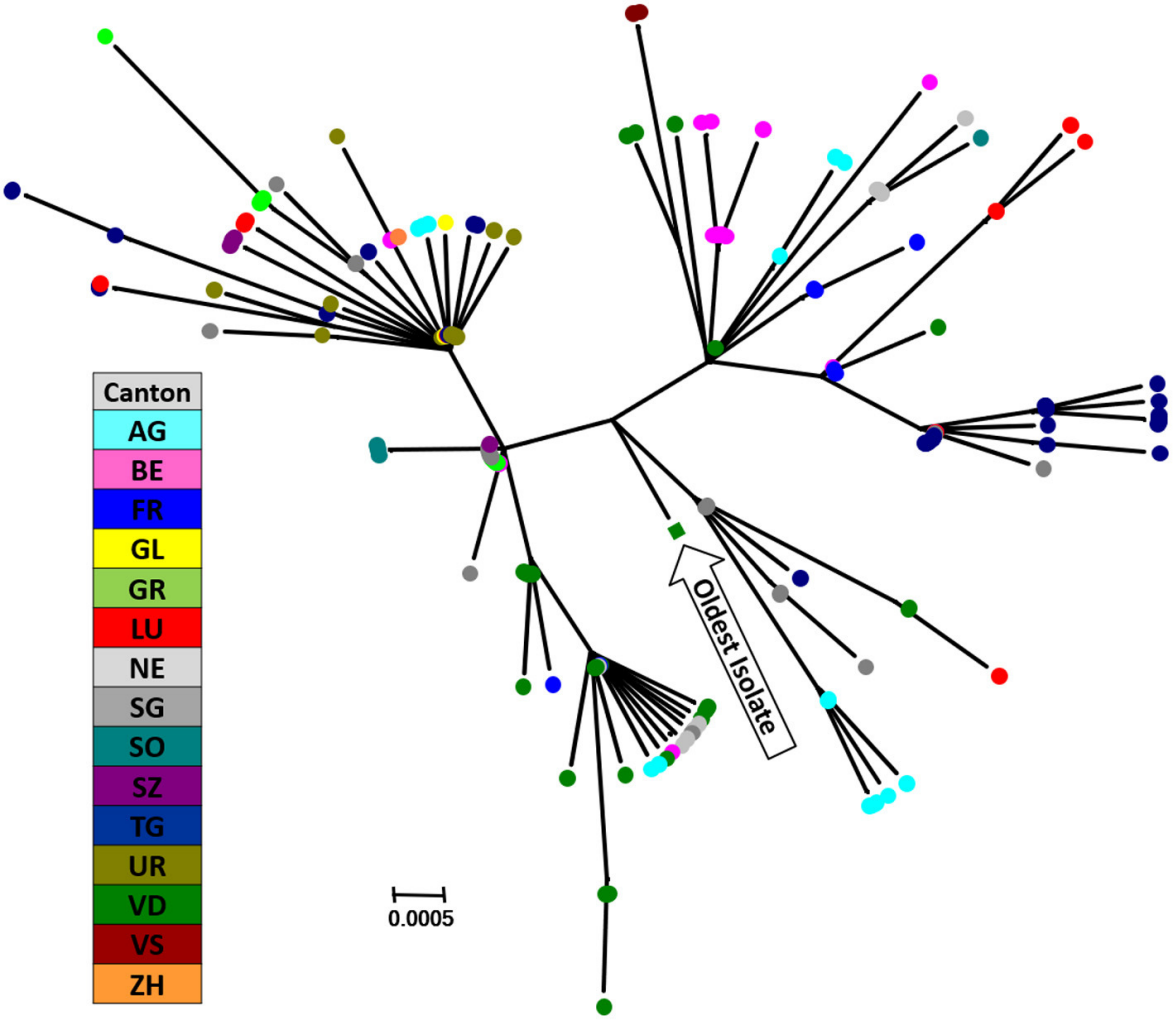
Phylogenetic analysis of an alleged infection chain analysing samples with identical sequences in the 5-UTR. Fragments of 978 bp in the NS2-3 region of the viral genome were sequenced and are shown in a phylogenetic tree. Each circle represents one single sequence from a PI animal sampled between May 2015 and February 2019, with different colours per canton representing the place of birth of the PI animal. The most antecedent sample within this cluster is indicated. The evolutionary history was inferred by using the Maximum Likelihood method based on the Kimura 2-parameter model (184), and the tree with the highest log likelihood is shown. A discrete gamma distribution was used to model evolutionary rate differences among sites. The tree is drawn to scale, with branch lengths measured in the number of substitutions per site. There are maximally 11 nucleotide difference between these samples (median = 5). The analysis involved 214 nucleotide sequences. Codon positions included were 1st + 2nd + 3rd + non-coding. There were a total of 927 positions in the final dataset. Evolutionary analyses were conducted in MEGA7 (18). The nucleotide sequences used were submitted to GenBank, accession no. MW936384—MW936597.

Currently, there appears to be around 10 chains of infection remaining, with some of them circulating for several years. The largest cluster we observed, i.e., isolates with identical sequences in the short stretch of the 5′-UTR, contains samples from around 1,000 animals collected since 2011. With pestiviruses being RNA viruses with a considerable mutation rate, it is, however, not plausible that all these isolates represent identical viruses, albeit a common origin cannot be excluded. Thus, the rather low resolution in the 5′-UTR is clearly sufficient to allocate the sequences to a specific (sub-)genotype, but it is insufficient to differentiate individual virus isolates. To enhance the resolution in sequencing to be of help for molecular epidemiology, we established a pilot scheme where we sequenced fragments of 800–1,000 nucleotides in length of selected clusters with identical sequences in the 5′-UTR by classical Sanger sequencing. This study confirmed that BVD viral strains can be further differentiated using these larger fragments, as exemplified in Figure 4. This differentiation requires the analysis of regions much more heterogeneous than the 5′-UTR, which made it unfeasible to design a single PCR-primer pair for all virus strains. This will clearly increase the costs, despite using well-established, cost-effective Sanger sequencing. In addition, data editing and interpretation are much more elaborate, which will considerably increase hands-on time required for analysis. Currently, this extended analysis cannot be performed on a routine basis in Switzerland with the available resources. Nonetheless, it might be a helpful tool in selected cases to support the identification of a possible source of infection, or in the final stages of the eradication programme as similarly applied in Sweden (183) or Austria (78). However, this requires that samples from every PI animal identified nationwide are available for sequencing. But independent of the fact that molecular epidemiology is a useful mean in the identification of a source of infection, the crucial point in the eradication is and remains the factor “time,” i.e., the pace at which the source of infection can be identified and eliminated before the virus can further be transmitted.

## Summary and Outlook

Based on the rather high number of BVD antibody positive animals prior to 2008 (60), Switzerland decided to take a rather radical approach in testing all cattle within <1 year without prior testing of the herd seroprevalence, as was done in the Scandinavian counties (56). Together with the notion that vaccines were extremely rarely used in Switzerland, it was intended from the beginning that surveillance after initial testing for virus will be done by serology and, therefore, vaccination was prohibited from the start of the eradication scheme. Testing for virus in all newborn calves was performed until the end of 2012, when <0.02% of all calves born were PI, an impressive reduction in just 5 years after having started at roughly 1.4% in 2018 (59). By the end of 2020, 99.6% of all herds were declared BVD free, with only 42 herds out of ~34,000 farms housing cattle in the country (with ~43,000 farms in 2008 at the start of the eradication programme) and 105 farms with individual animals being locked, the latter being pregnant animals that might have been infected during pregnancy (“Trojan cows”). Such Trojan cows present a great risk for re-introduction of BVDV into previously naïve herds (119), and strict control of such animals is absolutely required. Overall, the following measures were most relevant to achieve eradication of BVDV in Swiss cattle: (i) Testing all animals in the first year to massively reduce the risk of infection, (ii) testing newborn calves within 5 days after birth and prompt elimination of PI animals, (iii) risk-based constraints on animal movement, (iv) nationwide uniform strategy including the ban on vaccination, (v) centrally organised data management, (vi) rigorous contact tracing of all PI animals identified, and (vi) last but not least, regular information and communication to all stakeholders to maintain high levels of motivation to achieve these goals.

Initially, it was assumed that it would take around 10 years for eradication to be completed, as was described for other countries (44). However, this assumption was obviously somewhat too optimistic as especially the final stages appear to be the crux of the eradication programme, and the current costs for the programme are higher than previously projected (52). In the last seven years, always more than 98.5% of the farms have already been BVD free, with a maximum of 99.8% at the end of 2014, but identifying and eliminating the last PI animals is the largest hurdle. The surveillance by serology is generally able to identify clusters of infection, but the time until the source of infection is finally identified and eliminated is probably too slow. The approach to trace all contacts of PI animals to identify and test animals at risk of infection proved to be not sufficiently effective to replace the surveillance of the complete population for virus by partial surveillance using antibody testing. Nevertheless, a high proportion of PI animals and even Trojan cows were identified by contact tracing very rapidly, indicating that a rigorous contact tracing is extremely useful to reduce the risk of infection. A final effort should now be taken to eradicate the virus from the few remaining farms applying a rather strict regime. This might be unfavourable for the few farms affected, but it would be of great benefit for the rest of the country, as some of these herds have continuing infection cycles over several years and regularly pose a risk of infection risk for all their contacts.

For the final achievement of BVD eradication in Switzerland, the following factors and measures are important for the programme to be successful, most of them already being in place:

- Consistent completion of the animal movement database by every user without any gaps, possibly applying more severe consequences for fraudulent entries.
- Continued strict application of biosecurity measures, incl. cattle trade and summer pasturing.
- Enhanced biosecurity measures and strict supervision and surveillance during calving of possible “Trojan dams”.
- Nationwide standardised procedure following positive results in antibody surveillance to achieve faster response across cantonal borders.
- Immediate start of investigations upon a positive result through antibody surveillance, if appropriate with coordination across cantonal borders. During these assessments, the role of transient infections and the fact that no test is 100% sensitive and specific must be taken into consideration.
- Shorten the time interval between active surveillance on the “farms of concern,” i.e., the few farms repetitively harbouring PI animals in recent years.
- Transfer of every virus positive sample to the reference laboratory for sequencing. Molecular epidemiology is a great tool to track chains of infection, but this is only of help if all sequence data are available nationwide.
- Separation of cattle and sheep. Where this is not feasible, voluntary sanitation of the sheep population concerned should be envisioned to avoid costly investigations over and over again.

The aforementioned measures should enable the identification if any remaining source of infection as quickly as possible, and to reduce the risk of further transmission of the virus to naïve pregnant animals within this time interval. A final, more rigorous effort for a rather short time might be required to achieve the final aim of eradicating BVDV from the cattle population in Switzerland. Nevertheless, even after successful completion of this task, continued surveillance needs to be implemented (i) as ruminant pestiviruses might be re-introduced into the highly susceptible cattle population, e.g., by animal import or contaminated semen or vaccines, and (ii) as pestiviruses remain endemic in small ruminants in Switzerland, mainly in sheep, and pose a constant risk for re-introduction. This surveillance scheme will also be a necessity for federal and European regulations to continuously report the freedom of disease (185), which will hopefully soon be achieved.

## Author Contributions

MS conceptualised the manuscript. MS and HSch wrote the first draught of the manuscript with helpful advice and input by AH, EDL, HSt, and MG. HSch, HSt, and MS prepared the figures. All authors edited the manuscript and have read and approved the final version.

## Conflict of Interest

The authors declare that the research was conducted in the absence of any commercial or financial relationships that could be construed as a potential conflict of interest.

## Publisher's Note

All claims expressed in this article are solely those of the authors and do not necessarily represent those of their affiliated organizations, or those of the publisher, the editors and the reviewers. Any product that may be evaluated in this article, or claim that may be made by its manufacturer, is not guaranteed or endorsed by the publisher.
