# Supplementary material for: Eradication of Bovine Viral Diarrhoea (BVD) in Cattle in Switzerland: Lessons Taught by the Complex Biology of the Virus
Source: Front Vet Sci. 2021 Sep 7;8:702730. doi: 10.3389/fvets.2021.702730 (PMC8452978; doi:10.3389/fvets.2021.702730)
Supplement: Supplementary file 1 [file Data_Sheet_1.PDF]

Eradication of bovine viral diarrhea (BVD) in cattle in Switzerland: Lessons taught by the complex biology of the virus

Matthias Schweizer[1,2*], Hanspeter Stalder[1,2], Anja Haslebacher[3], Martin Grisiger[4], Heinzpeter Schwermer[5] and Elena Di Labio[5]

[1] Institute of Virology and Immunology, Länggass-Str. 122, POB, 3001 Bern, Switzerland

[2] Department of Infectious Diseases and Pathobiology, Vetsuisse Faculty, University of Bern, Switzerland

[3] Veterinary Office Canton Solothurn, Hauptgasse 72, 4509 Solothurn, Switzerland

[4] Veterinärdienst der Urkantone, Föhneichstr. 15, 6440 Brunnen, Switzerland

[5] Federal Food Safety and Veterinary Office (FSVO), Schwarzenburgstrasse 155, 3003 Bern, Switzerland

Supplementary Material

Supplementary Table 1: GenBank accession numbers of all nucleotide sequences used in the phylogenetic analysis shown in Figure 1.

Accession no.	Isolate	Species*)
M96751.1	SD1	BVDV-1 (Pestivirus A)
KC853440.1	SuwaNcp	BVDV-1 (Pestivirus A)
NC_039237.1	890	BVDV-2 (Pestivirus B)
AB567658.1	Hokudai-Lab/09	BVDV-2 (Pestivirus B)
J04358.2	Alfort/Tuebingen	CSFV (Pestivirus C)
NC_002657.1	Strain Eystrup	CSFV (Pestivirus C)
MF102261.1	CH-R9336/11	BDV (Pestivirus D)
GU270877.1	H2121 (Chamois-1)	BDV (Pestivirus D)
MG770617.1	Ovine/IT/1756/17	Ovine pestivirus
MK618726.1	Ovine/IT/338710-3/2017	Ovine pestivirus
KM408491.1	Burdur/05-TR	Aydin-like (Pestivirus I)
NC_018713.1	Aydin/04-TR	Aydin-like (Pestivirus I)
KJ660072.1	PG-2	Giraffe (Pestivirus G)
NC_003678.1	Giraffe-1 H138	Giraffe (Pestivirus G)
JX985409.1	CH-KaHo/cont	HoBi-like (Pestivirus H)
NC_012812.1	Th/04_KhonKaen	HoBi-like (Pestivirus H)
NC_024018.2	Pronghorn antelope	Pronghorn (Pestivirus E)
NC_035432.1	Austria1	Linda pestivirus
MH807263.1	H482/E2/2014	Bungowannah (Pestivirus F)
NC_023176.1	Bungowannah	Bungowannah (Pestivirus F)
MK910228.1	PhoPeV_NS170386_B	Phocoena pestivirus
MK910227.1	PhoPeV_NS170385_L	Phocoena pestivirus
MK636874.1	DYAJ1	Pangolin pestivirus
MK636875.1	DYCS	Pangolin pestivirus
NC_025677.1	NrPV/NYC-D23	Rat (Pestivirus J)
KY370101.1	RtNn-PestV/HuB2014	Rat (Pestivirus J)
MK216752.1	GD-HJ-2017.04	APPV (Pestivirus K)
MN099167	CH-5620	APPV (Pestivirus K)
MH282908.1	BtSk-PestV-1/GX2017	Bat pestivirus

*) The classification or denomination of the species was done according to (1-4).

Supplementary Table 2: Summary of publications reporting experimental transient infections (TI) and the transmission to sentinel animals. Sentinel = number of sentinel animals involved; Infected = number of sentinel animals that got infected; Ratio = ratio of infected sentinel to total number of sentinel animals.

Sentinel [n]	Infected [n]	Ratio [%]	Virus	Remarks	Reference
6	0	0 %	BVDV-2 (RS886)		(5)
6	0	0 %	BVDV-2 (1373)		(5)
4	0	0 %	BVDV-2 (RS886)		(6)
4	0	0 %	BVDV-1b		(6)
2	0	0 %	BVDV-1 (Ho916)	Low dose	(7)
2	1	50 %	BVDV-1 (Ho916)	High dose	(7)
2	2	100 %	BVDV-1 (Ho916)	High dose & immunosuppression	(7)
4	0	0 %	BVDV-1		(8)
4	0	0 %	BVDV-1		(8)
14	0	0 %	BVDV-1		(9)
7	1	14 %	BVDV-1	3 TI animals	(10)
7	1	14 %	BVDV-2	3 TI animals	(10)
6	6	100 %	HoBi-like		(5)
62	5	8 %	BVDV-1/-2	Total (excl. HoBi-like)	
68	11	16 %	BVDV & HoBi	Total (incl. HoBi-like)	

Supplementary Table 3: Summary of publications reporting experimental persistent infections (PI) and the transmission to sentinel cattle. Sentinel = number of sentinel animals involved; Infected = number of sentinel animals that got infected; Ratio = ratio of infected sentinel to total number of sentinel animals.

Sentinel [n]	Infected [n]	Ratio [%]	Virus	Remarks	Reference
95	79	83 %	BVDV-1d	Feedlot, seroconversion in unvaccinated animals	(11)
13	4	31 %	BVDV-1	Indirect transmission	(12)
6	2	33 %	BVDV-1	Indirect transmission	(12)
6	6	100 %	BVDV-1b	PI heifer to young bulls	(13)
6	6	100 %	BVDV-2	PI heifer to young bulls	(13)
5	5	100 %	BVDV-1	Nose-to-nose contact for 6 hours	(9)
3	3	100 %	BVDV-1b	PI animal added after no TI transmission	(10)
5	5	100 %	HoBi-like	Also, transmission to goats, sheep, and pigs	(14)
18	9	50 %	BVDV-1d	Birth of PI from “Trojan cow”	(14)
152	114	75 %	BVDV-1/-2	Total (excl. HoBi-like)	
127	119	76 %	BVDV & HoBi	Total (incl. HoBi-like)	

References to Supplementary Tables:

1. Smith DB, Meyers G, Bukh J, Gould EA, Monath T, Muerhoff AS, et al. Proposed revision to the taxonomy of the genus *Pestivirus*, family *Flaviviridae*. *J Gen Virol* (2017) 98:2106-12. doi: 10.1099/jgv.0.000873
2. Jo WK, van Elk C, van de Bildt M, van Run P, Petry M, Jesse ST, et al. An evolutionary divergent pestivirus lacking the N^{pro} gene systemically infects a whale species. *Emerg Microbes Infect* (2019) 8:1383-92. doi: 10.1080/22221751.2019.1664940
3. Gao W-H, Lin X-D, Chen Y-M, Xie C-G, Tan Z-Z, Zhou J-J, et al. Newly identified viral genomes in pangolins with fatal disease. *Virus Evol* (2020) 6:veaa020. doi: 10.1093/ve/veaa020
4. Righi C, Petrini S, Pierini I, Giammarioli M, De Mia GM. Global distribution and genetic heterogeneity of border disease virus. *Viruses* (2021) 13:950. doi: 10.3390/v13060950
5. Ridpath JF, Falkenberg SM, Bauermann FV, VanderLey BL, Do Y, Flores EF, et al. Comparison of acute infection of calves exposed to a high-virulence or low-virulence bovine viral diarrhea virus or a HoBi-like virus. *Am J Vet Res* (2013) 74:438-42. doi: 10.2460/ajvr.74.3.438
6. Falkenberg SM, Dassanayake RP, Neill JD, Ridpath JF. Evaluation of bovine viral diarrhea virus transmission potential to naïve calves by direct and indirect exposure routes. *Vet Microbiol* (2018) 217:144-8. doi: 10.1016/j.vetmic.2018.03.012
7. Strong R, La Rocca SA, Paton D, Bensaude E, Sandvik T, Davis L, et al. Viral dose and immunosuppression modulate the progression of acute BVDV-1 infection in calves: evidence of long term persistence after intra-nasal infection. *PLoS ONE* (2015) 10:e0124689. doi: 10.1371/journal.pone.0124689
8. Niskanen R, Lindberg A, Tråvén M. Failure to spread bovine virus diarrhoea virus infection from primarily infected calves despite concurrent infection with bovine coronavirus. *Vet J* (2002) 163:251-9. doi: 10.1053/tvjl.2001.0657
9. Niskanen R, Lindberg A, Larsson B, Alenius S. Lack of virus transmission from bovine viral diarrhoea virus infected calves to susceptible peers. *Acta Vet Scand* (2000) 41:93-9. doi: 10.1186/BF03549659
10. Sarrazin S, Dewulf J, Mathijs E, Laureyns J, Mostin L, Cay AB. Virulence comparison and quantification of horizontal bovine viral diarrhoea virus transmission following experimental infection in calves. *Vet J* (2014) 202:244-9. doi: 10.1016/j.tvjl.2014.07.010
11. Fulton RW, Briggs RE, Ridpath JF, Saliki JT, Confer AW, Payton ME, et al. Transmission of Bovine viral diarrhea virus 1b to susceptible and vaccinated calves by exposure to persistently infected calves. *Can J Vet Res* (2005) 69:161-9.
12. Lindberg A, Stokstad M, Løken T, Alenius S, Niskanen R. Indirect transmission of bovine viral diarrhoea virus at calving and during the postparturient period. *Vet Rec* (2004) 154:463-7. doi: 10.1136/vr.154.15.463
13. Givens MD, Riddell KP, Edmondson MA, Walz PH, Gard JA, Zhang YJ, et al. Epidemiology of prolonged testicular infections with bovine viral diarrhea virus. *Vet Microbiol* (2009) 139:42-51. doi: 10.1016/j.vetmic.2009.04.029
14. Bauermann FV, Falkenberg SM, Decaro N, Flores EF, Ridpath JF. Experimental infection of calves, sheep, goats and pigs with HoBi-like viruses by direct inoculation or exposure to persistently infected calves. *Vet Microbiol* (2015) 181:289-93. doi: 10.1016/j.vetmic.2015.10.011
